# Supplementary material for: Analysis of the transcriptional logic governing differential spatial expression in Hh target genes
Source: PLoS One. 2019 Jan 7;14(1):e0209349. doi: 10.1371/journal.pone.0209349 (PMC6322776; doi:10.1371/journal.pone.0209349)
Supplement: S1 File — This file contains all the mathematical details of the theoretical analysis. Furthermore, it contains the discussion of the results that can be deduced for the single gradient case. (PDF) [file pone.0209349.s001.pdf]

SUPPLEMENTARY MATERIAL (S1)
Analysis of the transcriptional logic governing differential spatial expression in Hh target genes

M. Cambón, O. Sánchez

There are multiple cross-references between the main text and the supplementary material contained in this document. For this reason equation numbering in this Supplementary Material document continues the numbering in the main text. Bibliographical cites are independent in each document.

A Refining the regulation factor expressions

In this subsection we present the different procedures used in order to rewrite the regulation factor used in the BEWARE operator (9). We will work with two transcription factors: $[A]$ and $[R]$, with activation and repression interaction intensity constants a and r, and binding affinities determined by the dissociation constants K_A and K_R. The number of enhancers in the promoter will be denoted by n.

In the next paragraphs we will show a procedure for obtaining the sums:

$$S^{(n)}(x_A, x_R; \mathcal{C}) = \sum_{j_A, j_R \geq 0}^{j_A+j_R \leq n} C(\mathcal{C}) \frac{n!}{j_0! j_A! j_R!} x_A^{j_A} x_R^{j_R} \quad \text{being} \quad j_0 = n - j_A - j_R \tag{26}$$

appearing in regulation factor definition (10) by using the multinomial theorem:

$$(1 + x_A + x_R)^n = \sum_{j_0+j_A+j_R=n} \frac{n!}{j_0! j_A! j_R!} 1^{j_0} x_A^{j_A} x_R^{j_R} \quad \text{where} \quad j_0\,, j_A\,, j_R \geq 0\,. \tag{27}$$

Indeed, recalling expression (9), the BEWARE operator is written in terms of the regulation factor

$$F_{reg}([A],[R];\mathcal{C}) = \frac{\sum_{j_A, j_R \geq 0}^{j_A+j_R \leq n} C(\mathcal{C}) \frac{n!}{j_0! j_A! j_R!} \left(\frac{a[A]}{K_A}\right)^{j_A} \left(\frac{r[R]}{K_R}\right)^{j_R}}{\sum_{j_A, j_R \geq 0}^{j_A+j_R \leq n} C(\mathcal{C}) \frac{n!}{j_0! j_A! j_R!} \left(\frac{[A]}{K_A}\right)^{j_A} \left(\frac{[R]}{K_R}\right)^{j_R}} = \frac{S^{(n)}(aK_A^{-1}[A], rK_R^{-1}[R]; \mathcal{C})}{S^{(n)}(K_A^{-1}[A], K_R^{-1}[R]; \mathcal{C})}\,.$$

We will get several expression for the regulation factor depending on the cooperativity between TFs, i.e., non-cooperative (11), total-cooperative (12) and partial-cooperative (13), represented by $C(\mathcal{C})$. Next lines show the computations for the numerators of the regulation factors, for each cooperativity hypothesis, and the denominators will follow the same deduction imposing $a = r = 1$.

- **Regulation factor for non-cooperative species**

  In this case the $C(\mathcal{C}) = 1$ and by (27) the sum reads

$$\begin{aligned} & S^{(n)}(aK_A^{-1}[A], rK_R^{-1}[R]; \{A, R\}_1) \\ &= \sum_{j_A, j_R \geq 0}^{j_A+j_R \leq n} \frac{n!}{j_0! j_A! j_R!} \left(\frac{a[A]}{K_A}\right)^{j_A} \left(\frac{r[R]}{K_R}\right)^{j_R} = \sum_{j_0+j_A+j_R=n} \frac{n!}{j_0! j_A! j_R!} 1^{j_0} \left(\frac{a[A]}{K_A}\right)^{j_A} \left(\frac{r[R]}{K_R}\right)^{j_R} \\ &= (1 + aK_A^{-1}[A] + rK_R^{-1}[R])^n. \end{aligned}$$

- **Regulation factor for total-cooperative species**

  If the transcription factors cooperate between all of them, then the cooperativity function is described by (6). The cooperativity function makes a bit more difficult the calculus of the polynomial (27), and first we need to get rid of the cooperativity in both the numerator and denominator of the regulation factor.

This can be easily achieved by splitting the sum

$$S^{(n)}(aK_A^{-1}[A], rK_R^{-1}[R]; \{A,R\}_c) = \sum_{j_A, j_R \geq 0}^{j_A+j_R \leq n} \frac{n!}{j_0! j_A! j_R!} c^{(j_A+j_R-1)_+} \left(\frac{a[A]}{K_A}\right)^{j_A} \left(\frac{r[R]}{K_R}\right)^{j_R}$$

$$= 1 + \sum_{j_A, j_R \geq 1}^{j_A+j_R \leq n} \frac{n!}{j_0! j_A! j_R!} c^{(j_A+j_R-1)_+} \left(\frac{a[A]}{K_A}\right)^{j_A} \left(\frac{r[R]}{K_R}\right)^{j_R}$$

$$= 1 + \frac{1}{c} \sum_{j_A, j_R \geq 1}^{j_A+j_R \leq n} \frac{n!}{j_0! j_A! j_R!} \left(\frac{ca[A]}{K_A}\right)^{j_A} \left(\frac{cr[R]}{K_R}\right)^{j_R}$$

$$= 1 - \frac{1}{c} + \frac{1}{c} \sum_{j_A, j_R \geq 0}^{j_A+j_R \leq n} \frac{n!}{j_0! j_A! j_R!} \left(\frac{ca[A]}{K_A}\right)^{j_A} \left(\frac{cr[R]}{K_R}\right)^{j_R},$$

and using (27) as before

$$S^{(n)}(aK_A^{-1}[A], rK_R^{-1}[R]; \{A,R\}_c) = 1 - \frac{1}{c} + \frac{1}{c}\left(1 + ca\frac{[A]}{K_A} + cr\frac{[R]}{K_R}\right)^n.$$

- **Regulation factor for partial-cooperative species**

  If the TFs cooperate independently (eq. (7)), we can split the sum twice in the same way as in the total-cooperativity case, i.e.,

$$S^{(n)}(aK_A^{-1}[A], rK_R^{-1}[R]; \{\{A\}_{c_A}, \{R\}_{c_R}\}) = \sum_{j_A, j_R \geq 0}^{j_A+j_R \leq n} \frac{n!}{j_0! j_A! j_R!} c_A^{(j_A-1)_+} c_R^{(j_R-1)_+} \left(\frac{a[A]}{K_A}\right)^{j_A} \left(\frac{r[R]}{K_R}\right)^{j_R}$$

$$= \sum_{j_A, j_R \geq 1}^{j_A+j_R \leq n} \frac{n!}{j_0! j_A! j_R!} c_A^{(j_A-1)_+} c_R^{(j_R-1)_+} \left(\frac{a[A]}{K_A}\right)^{j_A} \left(\frac{r[R]}{K_R}\right)^{j_R} + \sum_{\substack{j_A \equiv 0 \\ j_R \geq 1}}^{j_R \leq n} \frac{n!}{j_0! j_R!} c_R^{(j_R-1)_+} \left(\frac{r[R]}{K_R}\right)^{j_R}$$

$$+ \sum_{\substack{j_A \geq 1 \\ j_R \equiv 0}}^{j_A \leq n} \frac{n!}{j_0! j_A!} c_A^{(j_A-1)_+} \left(\frac{a[A]}{K_A}\right)^{j_A} + 1$$

$$= \frac{1}{c_A c_R} \sum_{j_A, j_R \geq 1}^{j_A+j_R \leq n} \frac{n!}{j_0! j_A! j_R!} \left(\frac{c_A a[A]}{K_A}\right)^{j_A} \left(\frac{c_R r[R]}{K_R}\right)^{j_R} + \frac{1}{c_R} \sum_{\substack{j_A \equiv 0 \\ j_R \geq 1}}^{j_R \leq n} \frac{n!}{j_0! j_R!} \left(\frac{c_R r[R]}{K_R}\right)^{j_R}$$

$$+ \frac{1}{c_A} \sum_{\substack{j_A \geq 1 \\ j_R \equiv 0}}^{j_A \leq n} \frac{n!}{j_0! j_A!} \left(\frac{c_A a[A]}{K_A}\right)^{j_A} + 1$$

$$= \frac{1}{c_A c_R} \sum_{j_A, j_R \geq 0}^{j_A+j_R \leq n} \frac{n!}{j_0! j_A! j_R!} \left(\frac{c_A a[A]}{K_A}\right)^{j_A} \left(\frac{c_R r[R]}{K_R}\right)^{j_R} + \left(1 - \frac{1}{c_A}\right) \frac{1}{c_R} \sum_{\substack{j_A \equiv 0 \\ j_R \geq 1}}^{j_R \leq n} \frac{n!}{j_0! j_R!} \left(\frac{c_R r[R]}{K_R}\right)^{j_R}$$

$$+ \left(1 - \frac{1}{c_R}\right) \frac{1}{c_A} \sum_{\substack{j_A \geq 1 \\ j_R \equiv 0}}^{j_A \leq n} \frac{n!}{j_0! j_A!} \left(\frac{c_A a[A]}{K_A}\right)^{j_A} + 1 - \frac{1}{c_A c_R}$$

where, by the previous deduction,

$$S^{(n)}(aK_A^{-1}[A], rK_R^{-1}[R]; \{\{A\}_{c_A}, \{R\}_{c_R}\}) \tag{28}$$

$$= \frac{1}{c_A c_R}(1 + c_A a K_A^{-1}[A] + c_R r K_R^{-1}[R])^n + \left(1 - \frac{1}{c_R}\right) \frac{(1 + c_A a K_A^{-1}[A])^n}{c_A}$$

$$+ \left(1 - \frac{1}{c_A}\right) \frac{(1 + c_R r K_R^{-1}[R])^n}{c_R} + \left(1 - \frac{1}{c_A}\right)\left(1 - \frac{1}{c_R}\right).$$

B Transcriptional logics with null/total cooperativity

In this section we show some theoretical monotonicity properties of the BEWARE operator (14) that provide the mathematical justification of the features stated for the transcription logic in the case of opposing gradients and non/total cooperativity between TFs. In these results the analysed operators are in correspondence with the effects of the variations of affinity, total cooperativity, transcriptional activation/repression intensities and the number of binding sites. Firstly we show the following lemma:

Lemma C *Let us consider the function*

$$G(\delta) = \frac{\left(1-\frac{1}{c}\right)+\frac{1}{c}\left(1+a\frac{c[A]}{\delta K_A}+r\frac{c[R]}{\delta K_R}\right)^n}{\left(1-\frac{1}{c}\right)+\frac{1}{c}\left(1+c\frac{[A]}{\delta K_A}+c\frac{[R]}{\delta K_R}\right)^n} = \frac{\left(1-\frac{1}{c}\right)\delta^n+\frac{1}{c}\left(\delta+ac\frac{[A]}{K_A}+rc\frac{[R]}{K_R}\right)^n}{\left(1-\frac{1}{c}\right)\delta^n+\frac{1}{c}\left(\delta+c\frac{[A]}{K_A}+c\frac{[R]}{K_R}\right)^n}$$

where $[A],\ [R]\geq 0,\ K_A,\ K_R,\ a,\ r,\ \delta$ are positive real numbers, $n\geq 1$ is a natural exponent and c is a real constant bigger or equal to 1. Then G is decreasing with respect to δ if and only if $[A]$ and $[R]$ verify

$$a\frac{[A]}{\delta K_A}+r\frac{[R]}{\delta K_R}\geq \frac{[A]}{\delta K_A}+\frac{[R]}{\delta K_R},\ \textit{or equivalently } G(\delta)\geq 1\,, \tag{29}$$

and increasing if the reverse inequality holds.

Proof. This assertion can be easily verified computing

$$\frac{dG}{d\delta} = \frac{\left(1-\frac{1}{c}\right)\frac{n\delta^{n-1}}{c}\big(h(\beta)-h(\alpha)\big)+\frac{n}{c^2}\alpha^{n-1}\beta^{n-1}(\beta-\alpha)}{\Big(\left(1-\frac{1}{c}\right)\delta^n+\frac{1}{c}\beta^n\Big)^2}$$

where $\alpha=\delta+ac\frac{[A]}{K_A}+rc\frac{[R]}{K_R}$, $\beta=\delta+c\frac{[A]}{K_A}+c\frac{[R]}{K_R}$ and $h(z)=z^{n-1}(z-\delta)$. In the case of cooperativity ($c\geq 1$) the sign of this expression depends only on the differences $h(\beta)-h(\alpha)$ and $\beta-\alpha$. Indeed both differences take always the same sign since $\alpha,\beta>\delta$ and $h(z)$ is an strictly increasing function for $z\geq\delta$. Condition (29) is equivalent to $\beta<\alpha$ implying the negative character of $\frac{\partial G}{\partial\delta}$ while in the opposite case, $\beta>\alpha$, $\frac{\partial G}{\partial\delta}$ is positive by the previous considerations. □

This property of the BEWARE operator can be translated in the following biological terms: Proportional increments in the affinities (i.e., decrements in the K_i dissociation constants) give result to an increment of the activator and repressor signaling. Or in other terms, the cells that are in the repression region show a more repressed reaction to the signal (less transcription), and the same goes for the activators, showing a larger activated reaction (more transcription). A graphical representation of this effect can be seen in Panel B of S1 Fig.

On the other hand, the regulation factor is also monotonous with respect to total cooperativity, the number of enhancers, and the transcriptional activation/repression intensities.

Lemma D *Let us consider the function*

$$H(c,n,a,r) = \frac{\left(1-\frac{1}{c}\right)+\frac{1}{c}\left(1+ca\frac{[A]}{K_A}+cr\frac{[R]}{K_R}\right)^n}{\left(1-\frac{1}{c}\right)+\frac{1}{c}\left(1+c\frac{[A]}{K_A}+c\frac{[R]}{K_R}\right)^n} = \frac{c-1+\left(1+ca\frac{[A]}{K_A}+cr\frac{[R]}{K_R}\right)^n}{c-1+\left(1+c\frac{[A]}{K_A}+c\frac{[R]}{K_R}\right)^n}$$

where $[A],\ [R]\geq 0,\ K_A,\ K_R$ are positive real numbers and the variables verify $c,\ n\geq 1$, $a>1$ and $r<1$. Let us consider values $a,\ r,\ c,\ n,\ c_1\leq c_2,\ n_1\leq n_2,\ a_1\leq a_2$ and $r_1\leq r_2$. Then, H verifies

1. *$H(c_1,n_1,a,r)\leq H(c_2,n_2,a,r)$ if $[A]$ and $[R]$ verify $a\frac{[A]}{K_A}+r\frac{[R]}{K_R}\geq\frac{[A]}{K_A}+\frac{[R]}{K_R}$,*
2. *$H(c_1,n_1,a,r)\geq H(c_2,n_2,a,r)$ in the opposite case, that is, $a\frac{[A]}{K_A}+r\frac{[R]}{K_R}\leq\frac{[A]}{K_A}+\frac{[R]}{K_R}$,*
3. *and in any case $H(c,n,a_1,r_1)\leq H(c,n,a_2,r_2)$.*

Proof. In this case the partial derivative of the functional H with respect to c can be expressed as

$$\begin{aligned}\frac{\partial H}{\partial c} &= \frac{\Big(1+n\gamma\left(1+c\gamma\right)^{n-1}\Big)\Big(c-1+\left(1+c\epsilon\right)^n\Big)}{\left(c-1+\left(1+c\epsilon\right)^n\right)^2}-\frac{\Big(1+n\epsilon\left(1+c\epsilon\right)^{n-1}\Big)\Big(c-1+\left(1+c\gamma\right)^n\Big)}{\left(c-1+\left(1+c\epsilon\right)^n\right)^2}\,,\\ &= \frac{\left(c-1+\left(1+c\gamma\right)^n\right)}{\left(c-1+\left(1+c\epsilon\right)^n\right)}\big(g(\gamma)-g(\epsilon)\big)\end{aligned}$$

where $\gamma = a\frac{[A]}{K_A} + r\frac{[R]}{K_R}$, $\epsilon = \frac{[A]}{K_A} + \frac{[R]}{K_R}$. This derivative is positive if and only if the inequality $g(\gamma) > g(\epsilon)$ holds being $g(z) = \frac{1+nz(1+cz)^{n-1}}{c-1+(1+cz)^n}$ a monotone increasing function. In consequence, $\frac{\partial H}{\partial c}$ will be positive if and only if $\gamma > \epsilon$.

On the other hand, the partial derivative with respect to n can be calculated

$$\begin{aligned}\frac{\partial H}{\partial n} &= \frac{(1+c\gamma)^n\Big(c-1+(1+c\epsilon)^n\Big)\log(1+c\gamma) - (1+c\epsilon)^n\Big(c-1+(1+c\gamma)^n\Big)\log(1+c\epsilon)}{\big(c-1+(1+c\epsilon)^n\big)^2} \\ &= \frac{(c-1)\Big((1+c\gamma)^n\log(1+c\gamma) - (1+c\epsilon)^n\log(1+c\epsilon)\Big) + (1+c\gamma)^n(1+c\epsilon)^n\left(\log(1+c\gamma) - \log(1+c\epsilon)\right)}{\big(c-1+(1+c\epsilon)^n\big)^2}\end{aligned}$$

where we employ again previous notation. Clearly, this derivative is positive if and only if $\gamma > \epsilon$ thanks to the monotone increasing character of the functions $(1+z)^n\log(1+z)$ and $\log(1+z)$. Then by a sandwich argument we easily conclude the proof of *1.* and *2.*. The proof of *3.* is straightforward because parameters a and r appear only in the numerator of H. □

This property can be seen in the same biological terms as exposed in the last Lemma, where in this case increments in the total cooperation constant give result to an increment of the activator and repressor signaling. This is, cells that are in the repression region are more repressed, expressing less transcription, while the activated cells express a larger transcription (See Panel C in S1 Fig).

E Transcriptional logics with partial cooperativity between activators and repressors

In the previous section we have shown how variations of the (total) cooperativity induced a global modulation of the signal. This is, both repressors and activators enhanced their response to the signal, and the transcription rate of the protein increased in regions where the cells are active and decreased there where the cells were repressed. However, if the cooperativity is not symmetric and we consider transcription factors that cooperate partially (i.e., activators with activators or repressors with repressors), the effect over the transcription loses also its symmetric behaviour such that:

- If only the repressors cooperate between them (i.e., $c_R > 1$ and $c_A = 1$), then the transcription rate will decrease the more c_R increases.
- If only the activators cooperate between them (i.e., $c_A > 1$ and $c_R = 1$), then the transcription rate will increase the more c_A increases.

This monotonous behaviour can be read in the following lemma:

Lemma F *Let us consider the function*

$$H_p(c_A, c_R, a, r) = \frac{(1+ac_A\tilde{A}+rc_R\tilde{R})^3 + (c_A-1)(1+rc_R\tilde{R})^3 + (c_R-1)(1+ac_A\tilde{A})^3 + (c_A-1)(c_R-1)}{(1+c_A\tilde{A}+c_R\tilde{R})^3 + (c_A-1)(1+c_R\tilde{R})^3 + (c_R-1)(1+c_A\tilde{A})^3 + (c_A-1)(c_R-1)}$$

where $c_A,\ c_R \geq 1$, $a > 1$ *and* $r < 1$.
Let us consider values $c_{A1} \leq c_{A2}$, $c_{R1} \leq c_{R2}$, $a_1 \leq a_2$, $r_1 \leq r_2$ *and some* $c_A \geq 1$, $c_R \geq 1$, $a \geq 1$ *and* $0 \leq r \leq 1$. *Then,* H_p *verifies*

1. $H_p(c_{A1}, 1, a, r) \leq H_p(c_{A2}, 1, a, r)$,
2. $H_p(1, c_{R1}, a, r) \geq H_p(1, c_{R2}, a, r)$,
3. $H_p(c_A, c_R, a_1, r_1) \leq H_p(c_A, c_R, a_2, r_2)$,

for all $\tilde{A}, \tilde{R} \geq 0$.

Proof.
Here we will compute the proof for item *2.*, noting that *1.* follows the same deduction.
Taking derivate with respect to c_R we deduce that

$$\operatorname{sign}\left(\frac{\partial H(1, c_R, a, r)}{\partial c_R}\right) = \operatorname{sign}\left\{(3r\tilde{R}\beta^2 + \alpha^3)(\bar{\beta}^3 + (c_R-1)\bar{\beta}^3) - (3\tilde{R}\bar{\beta}^2 + \bar{\alpha}^3)(\beta^3 + (c_R-1)\beta^3)\right\}$$

where
$$\beta = 1 + a\tilde{A} + rc_R\tilde{R}\,,\ \bar{\beta} = 1 + \tilde{A} + c_R\tilde{R}\,,\ \alpha = 1 + a\tilde{A}\ \text{ and } \bar{\alpha} = 1 + \tilde{A}\,.$$
Please note also that
$$\beta \geq \alpha\,,\ \alpha \geq \bar{\alpha}\,,\ \bar{\beta} \geq \bar{\alpha}\ \text{ and } \alpha\bar{\beta} \geq \bar{\alpha}\beta\,.$$
Now, multiplying by c_R the terms inside the sign function and doing some basic computations we get that the sign of the partial derivative will come from the sign of the function

$$\gamma_1 + (c_R - 1)\gamma_2\,,$$

where
$$\gamma_1 \equiv \left(3(\beta-\alpha)\beta^2 + \alpha^3\right)\bar{\beta}^3 - \left(3(\bar{\beta}-\bar{\alpha})\bar{\beta}^2 + \bar{\alpha}^3\right)\beta^3\,,$$
$$\gamma_2 \equiv \alpha^3\bar{\beta} - \bar{\alpha}^3\beta^3 + \left(3(\beta-\alpha)\beta^2 + \alpha^3\right)\bar{\alpha}^3 - \left(3(\bar{\beta}-\bar{\alpha})\bar{\beta}^2 + \bar{\alpha}^3\right)\alpha^3\,.$$
Now, taking into account the relations

$$X^3 - Y^3 = (X-Y)L_1(X,Y) = (X^2 - Y^2)L_2(X,Y)\,,$$

with
$$L_1(X,Y) = X^2 + Y^2 + XY\,, \tag{30}$$
and
$$L_2(X,Y) = \frac{X^2 + Y^2 + XY}{X+Y}\,, \tag{31}$$
then it can be easily checked that both γ_1 and γ_2 are negative, since they can be written as the product of a positive term times a negative term:

$$\gamma_1 \equiv (3\beta^2\bar{\beta}^2 - L_1(\alpha\bar{\beta}, \bar{\alpha}\beta))(\bar{\alpha}\beta - \alpha\bar{\beta}) \leq 0\,,$$
$$\gamma_2 \equiv (2L_2(\bar{\alpha}\beta, \alpha\bar{\beta}) - 3\alpha\bar{\alpha})(\bar{\alpha}^2\beta^2 - \alpha^2\bar{\beta}^2) \leq 0$$

by the properties that fullfill the functions β, α, $\bar{\beta}$ and $\bar{\alpha}$ presented at the beginning of the proof. This assures that the functional $H_p(1, c_R, a, r)$ is monotone decreasing with respect to c_R, and in particular if one takes $c_{R1} \leq c_{R2}$, then it is clear that

$$H_p(1, c_{R1}, a, r) \geq H_p(1, c_{R2}, a, r)\,.$$

Note also that item *3* can be easily proven by taking into account the monotonous behaviour of H_p with respect to both a and r, which is deduced by taking into account the direct dependence in the numerator of H_p's expression. □

Now, as we announced at the end of Section Methods, we develop the mathematical analysis of the function f defining the threshold determined by equation (20) when $n = 3$. Specifically we are going to:

- prove that the threshold is a regular increasing curve in the plane $[A] - [R]$ by using the implicit function theorem,
- describe the behaviour of this threshold,
- determine the dependence of the concentration boundaries $[A]_{th}$ with respect to proportional variations affinities between TFs and enhancers.

Imposing the threshold condition (20) we end up with the equivalent polynomial equation of the form
$$G\left(\frac{[A]}{K_A}, \frac{[R]}{K_R}\right) = 0 \tag{32}$$
being
$$\begin{aligned} G\left(\tilde{A}, \tilde{R}\right) \;&=\; \left(1 + c_A a\tilde{A} + c_R r\tilde{R}\right)^3 - \left(1 + c_A\tilde{A} + c_R\tilde{R}\right)^3 \\ &\quad + (c_R - 1)\left(\left(1 + c_A a\tilde{A}\right)^3 - \left(1 + c_A\tilde{A}\right)^3\right) + (c_A - 1)\left(\left(1 + c_R r\tilde{R}\right)^3 - \left(1 + c_R\tilde{R}\right)^3\right)\,. \end{aligned}$$

Please note that, by definition, G takes negative values in the repression region and positive values in the activation region.

We are going to prove that G fulfils the hypothesis of the implicit function theorem. With some basic calculations we rewrite the function G as a polynomial in the $\tilde{R} = \frac{[R]}{K_R}$ repression variable, and $\tilde{A} = \frac{[A]}{K_A}$ dependent coefficients,

$$G(\tilde{A}, \tilde{R}) \equiv P(\tilde{R}) = a_0(\tilde{A}) + a_1(\tilde{A})\tilde{R} + a_2(\tilde{A})\tilde{R}^2 + a_3(\tilde{A})\tilde{R}^3 \tag{33}$$

with

$$\begin{cases} a_0(\tilde{A}) &=& c_R\left((1 + ac_A\tilde{A})^3 - (1 + c_A\tilde{A})^3\right) \\ a_i(\tilde{A}) &=& \frac{3!}{(3-i)!i!}{c_R}^i\left(r^i(1 + ac_A\tilde{A})^{3-i} - (1 + c_A\tilde{A})^{3-i} + (c_A - 1)(r^i - 1)\right) \ \forall i = 1, 2, 3\,. \end{cases}$$

Here we state some lemmas allowing us to employ the implicit function theorem.

Lemma G *Let $a > 1$, $r < 1$ and $c_A, c_R \geq 1$. Then, for any positive value $\tilde{A}$, $P(\tilde{R})$ has an unique positive root, $\tilde{R}^*$, and $P'(\tilde{R}^*) = \frac{\partial G}{\partial \tilde{R}}(\tilde{A}, \tilde{R}^*) < 0$.*

Proof. First, note that $a_0 > 0$ and $a_3 = c_R^3 c_A(r^3 - 1) < 0$, due to the hypothesis on the parameters $a > 1$ and $r < 1$, and $\tilde{A} > 0$. Then, it is clear that

$$\lim_{\tilde{R}\to 0} P(\tilde{R}) = a_0 > 0 \qquad \text{and} \qquad \lim_{\tilde{R}\to\infty} P(\tilde{R}) = -\infty\,,$$

and hence there exist at least one positive root of $P(\tilde{R})$. Note also that, if P has no real extrema, then the result is trivially verified. If there exist real extrema of P, then their sign will provide information about the number of roots. In the cases of pairs of positive-negative and negative-negative extrema, it can be easily checked the existence of a unique positive root, verifying the result. In the remaining case, the existence of two positive extrema would imply the existence of three positive roots. We are going to prove that this case cannot be achieved with the conditions of the parameters that the polynomial works with.

The hypothesis of two real positive extrema would imply

$$\begin{cases} \tilde{R}_0^{(+)} > 0 \iff -a_2 + \sqrt{a_2^2 - 3a_3a_1} < 0\,, \\ \tilde{R}_0^{(-)} > 0 \iff -a_2 - \sqrt{a_2^2 - 3a_3a_1} < 0\,, \end{cases} \quad \text{being} \quad \tilde{R}_0^{\pm} = \frac{-a_2 \pm \sqrt{a_2^2 - 3a_3a_1}}{3a_3}\,, \tag{34}$$

due to $a_3 < 0$ and assuming $a_2^2 - 3a_3a_1 \geq 0$. For the same reason $\tilde{R}_0^{(-)} \geq \tilde{R}_0^{(+)}$ and in consequence condition (34) can be equivalently written as

$$\sqrt{a_2^2 - 3a_3a_1} < a_2\,. \tag{35}$$

From (35) we easily deduce that

$$a_2 > 0 \tag{36}$$

and

$$a_1 < 0 \tag{37}$$

are necessary conditions because $a_3 < 0$. Let us prove that both conditions, (36) and (37), are not compatible and in consequence (34) can not be verified.

(36) can be written equivalently as $(r^2a - 1)\tilde{A} + r^2 - 1 > 0$, which holds if and only if

$$r^2a > 1 \quad \text{and} \quad \tilde{A} > \frac{1 - r^2}{r^2a - 1} \tag{38}$$

are simultaneously fulfilled.

On the other hand, condition (37) can be explicitly expressed as

$$(ra^2 - 1)c_A\tilde{A}^2 + 2(ra - 1)\tilde{A} + r - 1 < 0\,.$$

In particular, when $r^2a > 1$, this inequality requieres $2(ra - 1)\tilde{A} + r - 1 < 0$ which occurs if and only if

$$\tilde{A} < \frac{1 - r}{2(ra - 1)}. \tag{39}$$

Now, let us observe that necessary conditions (38) and (39), respectively for (36) and (37), are not compatible at all since $r < 1$, $r^2a > 1$ and thus:

$$\frac{1 - r^2}{r^2a - 1} > \frac{1 - r^2}{2(r^2a - 1)} > \frac{1 - r}{2(ra - 1)}\,.$$

□

In an absolutely symmetric manner we can prove the analogous result fixing the variable $\tilde{R}$ and the corresponding polynomial $\bar{P}(\tilde{A}) = G(\tilde{A}, \tilde{R})$.

Lemma H *Let $a > 1$, $r < 1$ and $c_A, c_R \geq 1$. Then, for any positive value $\tilde{R}$, $\bar{P}(\tilde{A})$ has an unique positive root, $\tilde{A}^*$, and $\bar{P}'(\tilde{A}^*) = \frac{\partial G}{\partial \tilde{A}}(\tilde{A}^*, \tilde{R}) > 0$.*

Both results allow us to define a bijective function such that $f(\tilde{A}) = \tilde{R}^*$ and $f^{-1}(\tilde{R}) = \tilde{A}^*$ because of the uniqueness of the roots of $P(\tilde{R})$ and $\bar{P}(\tilde{A})$. The implicit function theorem gives that f is regular and increasing, since, $G(\tilde{A}, f(\tilde{A})) = 0$ for all $\tilde{A} > 0$ then

$$0 = \frac{\partial G}{\partial \tilde{A}}(\tilde{A}, f(\tilde{A})) + \frac{\partial G}{\partial \tilde{R}}(\tilde{A}, f(\tilde{A}))f'(\tilde{A}) = \bar{P}'(\tilde{A}) + P'(f(\tilde{A}))f'(\tilde{A}) \quad \Longrightarrow \quad f'(\tilde{A}) = -\frac{\bar{P}'(\tilde{A})}{P'(f(\tilde{A}))} > 0\,. \qquad (40)$$

Indeed, the threshold could be computed explicitly by applying the classical Tartaglia-Cardano's method (see [1] Section 3.8.). However, we can show without using these explicit expressions that f tends asymptotically to a straight line as the concentration of the TFs increases. This can be easily shown by simply evaluating the threshold condition (32) on $\tilde{R} = f(\tilde{A})$ and dividing by $\tilde{A}^3$ the whole equation. Then, tending the activators concentration to infinity leads to the equation

$$\begin{aligned}
&\lim_{\tilde{A}\to\infty} && \left(c_A a + c_R r \frac{f(\tilde{A})}{\tilde{A}}\right)^3 - \left(c_A + c_R \frac{f(\tilde{A})}{\tilde{A}}\right)^3 + (c_R - 1)\left((c_A a)^3 - c_A^3\right) \\
&+ && (c_A - 1)\left(\left(c_R r \frac{f(\tilde{A})}{\tilde{A}}\right)^3 - \left(c_R \frac{f(\tilde{A})}{\tilde{A}}\right)^3\right) \\
&= && (c_A a + c_R r\alpha)^3 - (c_A + c_R\alpha)^3 + (c_R - 1)\left((c_A a)^3 - c_A^3\right) + (c_A - 1)\left((c_R r\alpha)^3 - (c_R\alpha)^3\right) = 0\,,
\end{aligned}$$

where $\alpha = \lim_{\tilde{A}\to\infty} \frac{f(\tilde{A})}{\tilde{A}}$ is the slope that needs to be finite in the limit in order to fullfill the equation, and hence in the limit $f'(\tilde{A}) \to \alpha$.

Indeed, one can compare this threshold with (15) by simply evaluating G in the straight line (15) since

$$G\left(\tilde{A}, \frac{1-a}{r-1}\tilde{A}\right) = \bar{P}(\tilde{A}) = (\bar{a}_2 + \bar{a}_3\tilde{A})\tilde{A}^2\,,$$

where the coefficients $\bar{a}_2$ and $\bar{a}_3$ are given by (22). This convoluted relation between the parameters defines the activation-repression range compared to the linear threshold, where depending on the sign of the coefficients we get:

- If $\bar{a}_2 > 0$ and $\bar{a}_3 > 0$, then the threshold for the BEWARE operator with partial cooperativity is over the threshold (15). In consequence, the activation range will increase due to partial cooperativity.
- If $\bar{a}_2 < 0$ and $\bar{a}_3 < 0$, then the threshold for the BEWARE operator with partial cooperativity is under the threshold (15). In consequence, the activation range will decrease due to partial cooperativity.
- If $\bar{a}_2 < 0$ and $\bar{a}_3 > 0$, the threshold for the the BEWARE operator with partial cooperativity is over the threshold (15) if $\tilde{A} = \frac{[A]}{K_A} < \frac{-\bar{a}_2}{\bar{a}_3}$ and is under (15) otherwise. Under hypothesis (18) the change in the activation range will be determined explicitly by the total level of Ci protein in the system, h. Let us consider
$$[A]^l_{th} = \frac{h}{\frac{a-1}{1-r}\frac{K_R}{K_A} + 1}$$
the intersection point between (15) and (18) and $[A]_{th}$ the intersection point between the threshold for the BEWARE operator with partial cooperativity. Under hypothesis (17) this expression can not be computed explicitly.
Then, if $[A]^l_{th} < \frac{-\bar{a}_2}{\bar{a}_3}K_A$ it can be easily checked that $[A]_{th} < [A]^l_{th}$. On the other hand, when, $[A]^l_{th} > \frac{-\bar{a}_2}{\bar{a}_3}K_A$ the reverse inequality holds $[A]_{th} > [A]^l_{th}$. That is, the activation range is larger or shorter with partial cooperativity depending on h. If $[A]^l_{th} = \frac{-\bar{a}_2}{\bar{a}_3}K_A$ the threshold is given by (15).
- If $\bar{a}_2 > 0$ and $\bar{a}_3 < 0$, the situation is exactly opposite to the previous one. Now, if $[A]^l_{th} > \frac{-\bar{a}_2}{\bar{a}_3}K_A$ then $[A]_{th}$ verifies $\frac{-\bar{a}_2}{\bar{a}_3}K_A < [A]_{th} < [A]^l_{th}$ and the activation range will increase. Furthermore, if $[A]^l_{th} < \frac{-\bar{a}_2}{\bar{a}_3}K_A$ then the activation range will decrease because $\frac{-\bar{a}_2}{\bar{a}_3}K_A > [A]_{th} > [A]^l_{th}$ holds and in the case of equality $[A]^l_{th} = \frac{-\bar{a}_2}{\bar{a}_3}K_A$ the activation range remains the same.

Now we are going to discuss about the monotonicity properties of the activator concentration thresholds given by (25) with respect to proportional variations of affinities.

Lemma I *Let us consider $[A]_{th} \equiv [A]_{th}(\delta)$ the implicit solution to the equation*

$$[A]_{th} + \delta K_R f\left(\frac{[A]_{th}}{\delta K_A}\right) = h(x) \tag{41}$$

where K_A, K_R are fixed positive real numbers, f is the threshold for a BEWARE operator with partial cooperativity (determined by constants a, r, c_A and c_R) and δ a positive real parameter. Then, $[A]_{th}$ is increasing with respect to δ if

$$f\left(\frac{[A]_{th}}{\delta K_A}\right) - f'\left(\frac{[A]_{th}}{\delta K_A}\right)\frac{[A]_{th}}{\delta K_A} > 0 \tag{42}$$

and monotone decreasing otherwise. In consequence, when f is strictly concave (respectively convex) $[A]_{th}$ is strictly monotone increasing (respectively decreasing).

Proof. From (17), we have a curve in the $[A]-[R]$ plane of the form $\vec{\gamma}(x) = ([A](x), [R](x))$. This curve, from (16), can be reparametrized by $[A]$ such as $\vec{\gamma}([A]) = ([A], \phi([A]))$. It is important to note that $\phi([A])$ is a decreasing function since

$$\phi'([A]) = R'([A]^{-1}([A]))\frac{1}{[A]'([A])} < 0.$$

Note also that $\phi([A])$ is a straight line under the conservation of the total concentration condition (18). Then, by combining (41) and (17), we get

$$\delta K_R f\left(\frac{[A]_{th}}{\delta K_A}\right) = \phi([A]_{th})$$

and its derivative leads to

$$K_R f\left(\frac{[A]_{th}}{\delta K_A}\right) - f'\left(\frac{[A]_{th}}{\delta K_A}\right)\frac{K_R[A]_{th}}{\delta K_A} = \left(-\frac{K_R}{K_A}f'\left(\frac{[A]_{th}}{\delta K_A}\right) + \phi'([A]_{th})\right)\frac{d[A]_{th}}{d\delta}\,,$$

from where we conclude that the sign of $\frac{d[A]_{th}}{d\delta}$ comes from the opposite sign of

$$f\left(\frac{[A]_{th}}{\delta K_A}\right) - f'\left(\frac{[A]_{th}}{\delta K_A}\right)\frac{K_R}{\delta K_A}$$

since $f' > 0$ and $\phi' < 0$.

□

Lemma J *In the case of cooperative repressors $[A]_{th}$ defined in (41) is decreasing with respect to δ, and increasing in the case of cooperative activators.*

Proof. Here we will deduce the proof for the case of partial cooperativity between repressors (the activator case follows the same procedure).

Rewriting inequation (42) in terms of (40) and using Lemma G, we can equivalently check that

$$\frac{\partial G}{\partial \tilde{A}}\tilde{A} + \frac{\partial G}{\partial \tilde{R}}\tilde{R} < 0\,, \tag{43}$$

where for simplicity we have noted $f\left(\frac{[A]_{th}}{\delta K_A}\right) = \tilde{R}$ and $\frac{[A]_{th}}{\delta K_A} = \tilde{A}$.

Imposing $c_A = 1$ and $c_R > 1$ it can be easily checked that the inequation (43) is equivalent to

$$(1 + a\tilde{A} + rc_R\tilde{R})^2 - (1 + \tilde{A} + c_R\tilde{R})^2 + (c_R - 1)\left((1 + a\tilde{A})^2 - (1 + \tilde{A})^2\right) > 0\,. \tag{44}$$

Please note that the lefthand side of this inequation coincides with the expression of $G(\tilde{A}, \tilde{R})$ in the repressors partial cooperative case with exponents equal to 2. Taking into account that $(\tilde{A}, \tilde{R})$ are points of the threshold, we get

$$\begin{aligned} 0 &= G(\tilde{A}, \tilde{R}) = (1 + a\tilde{A} + rc_R\tilde{R})^3 - (1 + \tilde{A} + c_R\tilde{R})^3 + (c_R - 1)\left((1 + a\tilde{A})^3 - (1 + \tilde{A})^3\right) \\ &= \left((1 + a\tilde{A} + rc_R\tilde{R})^2 - (1 + \tilde{A} + c_R\tilde{R})^2\right)L_2(1 + a\tilde{A} + rc_R\tilde{R}, 1 + \tilde{A} + c_R\tilde{R}) \\ &\quad + (c_R - 1)\left((1 + a\tilde{A})^2 - (1 + \tilde{A})^2\right)L_2(1 + a\tilde{A}, 1 + \tilde{A}) \\ &= \left((1 + a\tilde{A} + rc_R\tilde{R})^2 - (1 + \tilde{A} + c_R\tilde{R})^2\right) + (c_R - 1)\frac{\left((1 + a\tilde{A})^2 - (1 + \tilde{A})^2\right)L_2(1 + a\tilde{A}, 1 + \tilde{A})}{L_2(1 + a\tilde{A} + rc_R\tilde{R}, 1 + \tilde{A} + c_R\tilde{R})}\,. \end{aligned} \tag{45}$$

where $L_2(x,y)$ is defined at (31). From (45) we deduce the chain of inequalities

$$1+\tilde{A} \leq 1+a\tilde{A} \leq 1+a\tilde{A}+rc_R\tilde{R} \leq 1+\tilde{A}+c_R\tilde{R}$$

which allow us to get

$$\frac{L_2(1+a\tilde{A},1+\tilde{A})}{L_2(1+a\tilde{A}+rc_R\tilde{R},1+\tilde{A}+c_R\tilde{R})} \leq 1$$

thanks to the monotonic behaviour of $L_2(x,y)$ with respect to x and y. Hence (44) holds. □

See S2 and S3 Figs for a graphical example of how cooperative activators and repressors fulfil this condition.

K Transcription logic in the case of single gradients

In next paragraphs we show how our analysis can be adapted in the case of genes controlled by a single gradient of transcription factors, systems where the transcription logic is well known. Although the deduction of the BEWARE operators is valid in the presence of two TFs we can get the expression of the transcription rates simply by imposing in the deducted expressions null concentrations of the non existent TF, that is, $[R]=0$ for single activator gradients, and $[A]=0$ for single repressor gradients. In this case, all the versions (10), (11), (12) and (13) end up in the expressions

$$F_{reg}([A];\{A\}_c) = \frac{1-\frac{1}{c}+\frac{1}{c}\left(1+ac\frac{[A]}{K_A}\right)^n}{1-\frac{1}{c}+\frac{1}{c}\left(1+c\frac{[A]}{K_A}\right)^n} \tag{46}$$

and

$$F_{reg}([R];\{R\}_c) = \frac{1-\frac{1}{c}+\frac{1}{c}\left(1+rc\frac{[R]}{K_R}\right)^n}{1-\frac{1}{c}+\frac{1}{c}\left(1+c\frac{[R]}{K_R}\right)^n} \tag{47}$$

for the single activator gradient and the single repressor gradient respectively. Here $c=1$ in the non cooperative case and $c>1$ if total cooperation holds. Partial cooperation does not make sense in these cases.

These expressions allow us to theoretically justify, for instance, that in the case of activators high-affinity binding sites causes a broader gene expression. Let us assume two hypothetic genes, *gene1* and *gene2* activated by the same activator gradient, A. However, binding sites for *gene2* exhibit higher affinity that binding sites for *gene1* for the same molecules of A. This fact in our notation implies

$$K_A^{g2} = \delta K_A^{g1} < K_A^{g1} \quad \text{since } \delta << 1\,. \tag{48}$$

where K_A^* denote dissociation constants for the gene $*$. Then, the monotonicity property stated in Lemma C gives that

$$F_{reg}^{g2}([A];\{A\}_c) = \frac{1-\frac{1}{c}+\frac{1}{c}\left(1+ac\frac{[A]}{K_A^{g2}}\right)^n}{1-\frac{1}{c}+\frac{1}{c}\left(1+c\frac{[A]}{K_A^{g2}}\right)^n} > \frac{1-\frac{1}{c}+\frac{1}{c}\left(1+ac\frac{[A]}{K_A^{g1}}\right)^n}{1-\frac{1}{c}+\frac{1}{c}\left(1+c\frac{[A]}{K_A^{g1}}\right)^n} = F_{reg}^{g1}([A];\{A\}_c)$$

because $ac\frac{[A]}{K_A^{g2}} > c\frac{[A]}{K_A^{g2}}$ holds whenever $[A]>0$ since $a>1$. That is, by formula (9) any cell under the same activator concentration will express *gene2* with a higher rate than the expression rate of *gene1*. In Table L.this fact has been described by indicating that a decrement in affinity (↓TFs affinity) decreases the activation efficiency (↓Act) and in consequence it will be observed a lower cellular expression range (↓CER).

This conclusion is reflected in Table L, column **a)**, where ↓Act denotes the global attenuation of the activating signal along the imaginal disc and ↓CER denotes the reduction in the cellular expression ranges (CERs) caused by the signal modulation. In the case of a single repressor gradient the pattern is analogous but with inverse consequences, see Table L, column **b)**. In both cases the signal role is clear: it enhances the activation or repression along all over the imaginal disc. The differences in affinity and cooperativity modify the gene expression by modulating the signal, that is, weakening or reinforcing the activation or repression wherever the single gradient is present. Example graphs can be found in S4 Fig.

The same conclusions can be extracted from monotonicity properties stated in Lemma D in the case of decrements

- of cooperativity between activators (↓TFs-TFs coop) given by $c_1 < c_2$,
- number of enhancers (no. enhancers),

- or interaction intensity (↓TFA- RNAP coop.) expressed in terms of the cooperativity between the activator and the RNA polymerase ($a_1 < a_1$).

In the case of a single repressor gradient the monotonicity properties are exactly opposite because in the presence of repression $rc\frac{[R]}{K_R^*} > c\frac{[R]}{K_R^*}$ is always verified because $r < 1$. Thus, in this other case the same results show that the increments on affinity ($K_A^{g2} < K_A^{g1}$) , cooperativity between repressors ($c_1 < c_2$) , number of enhancers or interaction intensity ($r_1 > r_2$) intensify the repression signalling provoking smaller transcription rates. These results are summarised in Table L.

Biochemical characteristics	Single gradient **a)** Activator	**b)** Repressor
**1)** ↓ TFs affinity	↓Act ⇒ ↓CER	↓Rep ⇒ ↑CER
**2)** ↓ TFs-TFs coop.	↓Act ⇒ ↓CER	↓Rep ⇒ ↑CER
**3)** ↓ no. enhancers	↓Act ⇒ ↓CER	↓Rep ⇒ ↑CER
**4)** ↓ A-RNAP coop.	↓Act ⇒ ↓CER	∗ ∗ ∗
**5)** ↓ R-RNAP coop.	∗ ∗ ∗	↓Rep ⇒ ↑CER

Key: ↑ increase, ↓ decrease, ⇒ produces, ∗ ∗ ∗ not applicable, CER cellular expression range

Table L: **Transcriptional logic in the single TF gradient case.** The table shows how the differences in the biochemical characteristics in the presence of single gradients (columns **a)** and **b)**) always cause an decrement in activation and repression effects (↓Act, ↓Rep resp.). Thus the broader/narrower CERs are a consequence of globally higher/lower expression rates.

M Parameters

For the sake of biological reliability the values employed to generate black lines and the conserved TFs concentration in all the figures presented in this work have been adapted from fittings developed in [2] in the Drosophila system. The values employed in these figures are collected in Table N.

BEWARE constant	C_B	$1\,nM\text{min}^{-1}$
Transcriptional activation intensity	a	4.35
Transcriptional repression intensity	r	5×10^{-5}
Dissociation constant of activators for gene enhancers	K_A	$9 \times 10^1\,nM$
Dissociation constant of repressors for gene enhancers	K_R	$9 \times 10^1\,nM$
RNA polymerase binding affinity	K_{RP}	$[RNAP]$
RNA polymerase concentration	$[RNAP]$	K_{RP}
Total cooperativity constant	c	1
Activator partial cooperativity constant (cooperative activators)	c_A	10
Repressor partial cooperativity constant (cooperative activators)	c_R	1
Activator partial cooperativity constant (cooperative repressors)	c_A	1
Repressor partial cooperativity constant (cooperative repressors)	c_R	10
TFs total concentration	h	$24\,nM$
TFs gradient steepness	D	593

Table N: **Parameters used for the plots in black lines**. Please note that, for the single gradient case (S4 Fig), we only have taken into account the parameters of the corresponding TF gradient. The values for C_B, $[RNAP]$ and K_{RP} imply that the expression levels given by the BEWARE operators are bounded by $1nM/min$ being the basal level equal to $0.5nM/min$. In this way signal modulations can be properly appreciated.

Using Table N as a base, we have depicted with magenta lines in S1, S3 and S4 Figs the same systems with the following parameter variations:

- $c_R \to c_R \times 10^1$ (Higher R-R cooperativity)
- $c_A \to c_A \times 10^1$ (Higher A-A cooperativity)
- $K_A \to K_A \times 10^{-1}$ (Higher affinity)
- $K_R \to K_R \times 10^{-1}$ (Higher affinity)

References

[1] Abramovitz M., Stegun I.A. (eds.), *Handbook of mathematical functions (10ed)*, NBS, Washington D.C.;1972.

[2] Parker, D.S., White, M.A., Ramos, A.I., Cohen, B.A., Barolo, S., *The cis-Regulatory Logic of Hedgehog Gradient Responses: Key Roles for Gli Binding Affinity, Competition, and Cooperativity,* Sci. Signal. 2011;4: 1–16.
