# Supplementary material for: Analysis of the transcriptional logic governing differential spatial expression in Hh target genes
Source: PLoS One. 2019 Jan 7;14(1):e0209349. doi: 10.1371/journal.pone.0209349 (PMC6322776; doi:10.1371/journal.pone.0209349)
Supplement: S1 Fig — (PDF) [file pone.0209349.s002.pdf]

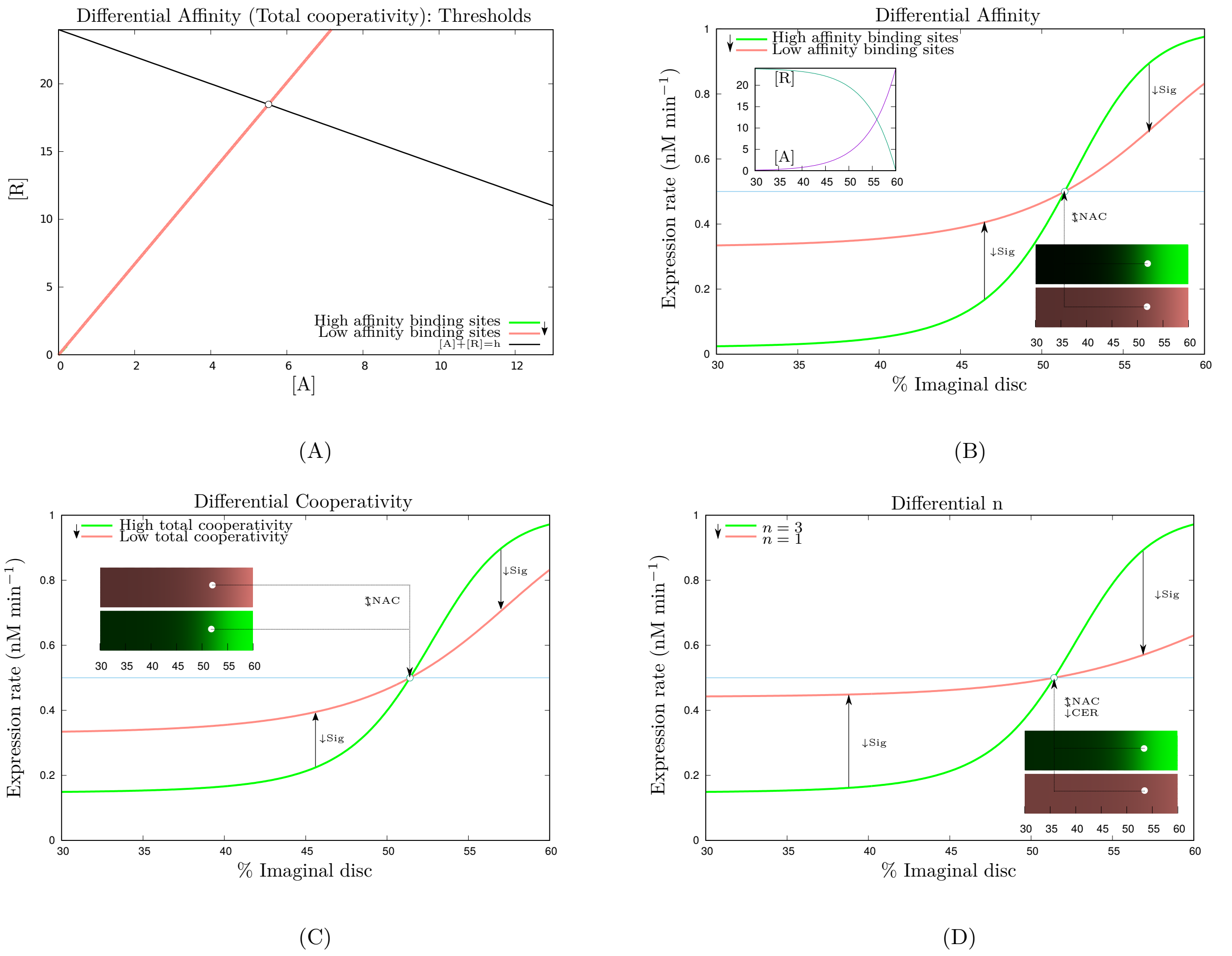

Fig S1: **Transcriptional logic in the presence of opposing gradients and non/total cooperativity (column a) of Table 1 of the main text)**.
Transcriptional effects of differences in three biochemical characteristics (affinities, cooperativity and number of enhancers). Note how the three mechanisms do not change the region of net active/repressed cells (basal expression level is at 0.5 indicated by light blue line), and cause a reduction of the activator and repressor signals. In Fig **(A)** we have depicted the threshold curve for this operator, where all the curves are the same since none of the mechanisms induce any change on the number of activated cells. The black line in Fig **(A)** gives the total TF concentration condition, (18), and black intersection circles denote the concentrations $[A]_{th}$ and $[R]_{th}$. These concentrations determine the spatial position of the limit of net differentiated activated region, indicated in **(B)**, **(C)** and **(D)** by blue circles. In order to better appreciate variations in the signal response we also include black-green and black-magenta bars to emulate the way that spatial expression is seen by fluorescent reporter imaging. Blue circles inside the bars indicate the position of a cell expressing at the basal level. The color scale used in these bars is shown in Fig 1**(C)** of the main text, where black color means no expression, and full color depicts high expression. The inset in **(B)** depicts the activator/repressors gradients that are present in the tissue. See Section E in S1 File for specific values.
