# Supplementary material for: Analysis of the transcriptional logic governing differential spatial expression in Hh target genes
Source: PLoS One. 2019 Jan 7;14(1):e0209349. doi: 10.1371/journal.pone.0209349 (PMC6322776; doi:10.1371/journal.pone.0209349)
Supplement: S3 Fig — (PDF) [file pone.0209349.s004.pdf]

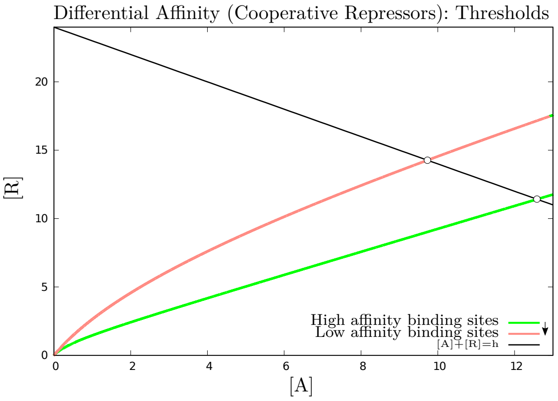

(A)

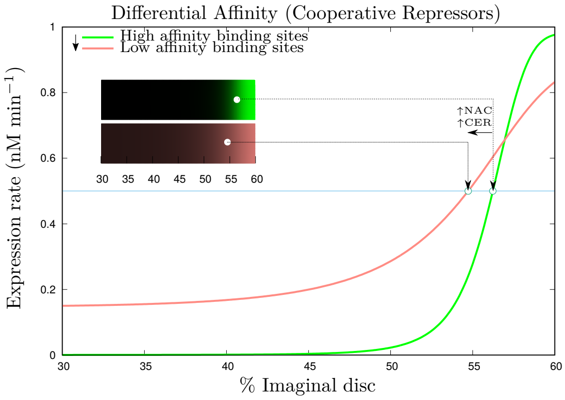

(B)

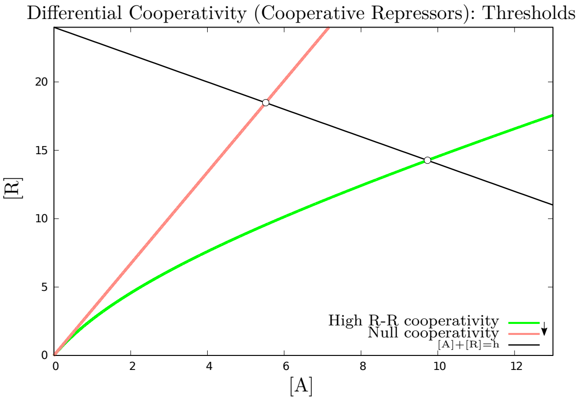

(C)

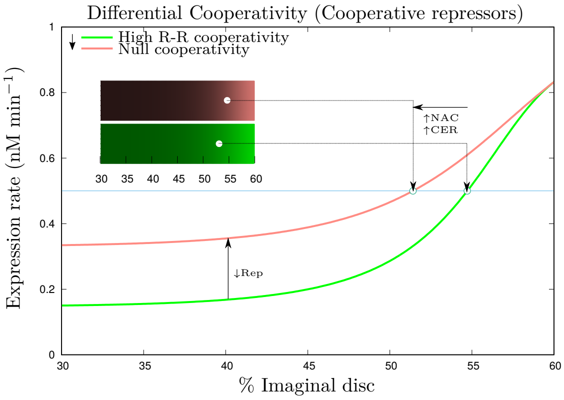

(D)

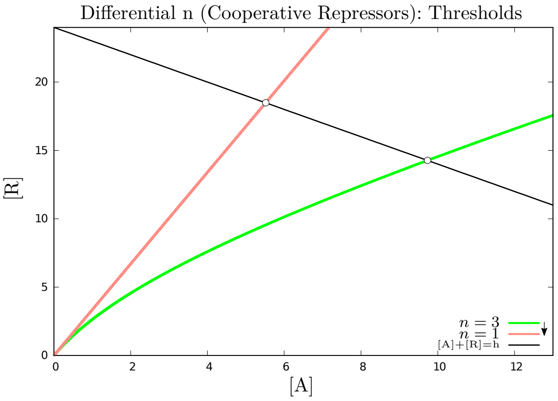

(E)

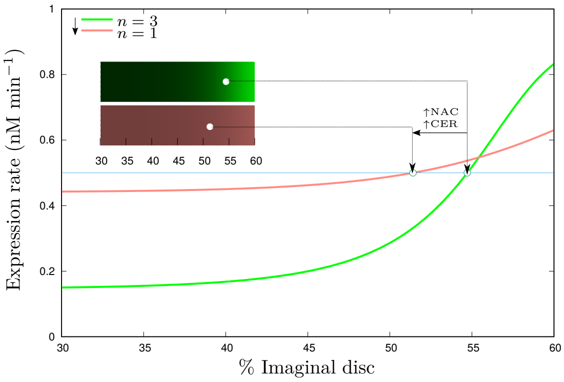

(F)

Fig S3: **Transcriptional logic in the presence of opposing gradients and partial cooperativity between repressors (column c) of Table 1 of the main text).**
Transcriptional effects coming from differences in three biochemical characteristics (affinity, cooperativity and number of enhancers). Figs **(A)**, **(C)** and **(E)** show the variation of the thresholds between activation/repression concentrations provoked by differences in affinity and cooperativity. Figs **(B)**, **(D)** and **(F)** show the corresponding change in the transcription rate, where both number of activated cells and cellular expression ranges increase due to the decrement of such mechanisms. For these graphs we have used the same keys as in S1 Fig.
