# Supplementary material for: Analysis of the transcriptional logic governing differential spatial expression in Hh target genes
Source: PLoS One. 2019 Jan 7;14(1):e0209349. doi: 10.1371/journal.pone.0209349 (PMC6322776; doi:10.1371/journal.pone.0209349)
Supplement: S4 Fig — (PDF) [file pone.0209349.s005.pdf]

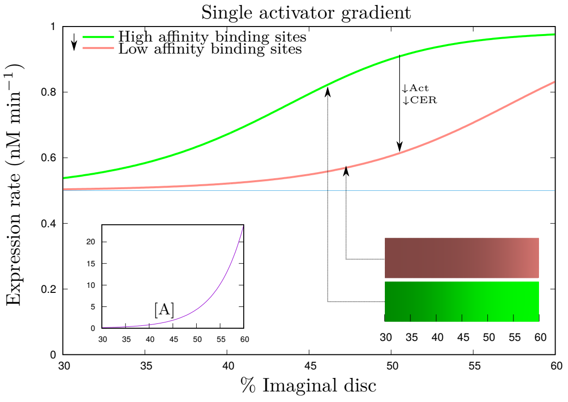

(A)

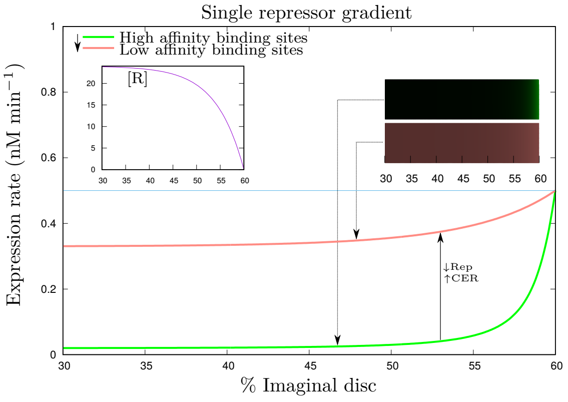

(B)

Fig S4: **Transcription logic in the case of single gradients. Columns a) and b) in Table A in S1 File**. Transcriptional effects of differences in affinity for binding sites. Note how decrements in affinity give different cellular expression depending on the TF gradient: in the presence of activator gradients the affinities decrement causes a total decrease in the expression rate, and hence less activation in the whole imaginal disc. On the contrary, if there is only a repressor gradient the response is completely different, giving rise to a higher expression rates in all the tissue (i.e., less total repression). For these graphs we have used the same keys as in S1 Fig.
